# Genomic and gene expression associations to morphology of a sexual ornament in the chicken

**DOI:** 10.1093/g3journal/jkac174

**Published:** 2022-07-08

**Authors:** Vid Bakovic, Andrey Höglund, Maria Luisa Martin Cerezo, Rie Henriksen, Dominic Wright

**Affiliations:** IFM Biology, University of Linköping, Linköping 581 83, Sweden; Science for Life Laboratory, Department of Environmental Science, Stockholm University, Stockholm 106 91, Sweden; IFM Biology, University of Linköping, Linköping 581 83, Sweden; IFM Biology, University of Linköping, Linköping 581 83, Sweden; IFM Biology, University of Linköping, Linköping 581 83, Sweden

**Keywords:** QTL, eQTL, chicken comb, domestication

## Abstract

How sexual selection affects the genome ultimately relies on the strength and type of selection, and the genetic architecture of the involved traits. While associating genotype with phenotype often utilizes standard trait morphology, trait representations in morphospace using geometric morphometric approaches receive less focus in this regard. Here, we identify genetic associations to a sexual ornament, the comb, in the chicken system (*Gallus gallus*). Our approach combined genome-wide genotype and gene expression data (>30k genes) with different aspects of comb morphology in an advanced intercross line (F8) generated by crossing a wild-type Red Junglefowl with a domestic breed of chicken (White Leghorn). In total, 10 quantitative trait loci were found associated to various aspects of comb shape and size, while 1,184 expression QTL were found associated to gene expression patterns, among which 98 had overlapping confidence intervals with those of quantitative trait loci. Our results highlight both known genomic regions confirming previous records of a large effect quantitative trait loci associated to comb size, and novel quantitative trait loci associated to comb shape. Genes were considered candidates affecting comb morphology if they were found within both confidence intervals of the underlying quantitative trait loci and eQTL. Overlaps between quantitative trait loci and genome-wide selective sweeps identified in a previous study revealed that only loci associated to comb size may be experiencing on-going selection under domestication.

## Introduction

Chickens have been domesticated for over 8,000 years, and in that time, a wide variety of different traits have been subject to selection, be it intentional or not. Large changes in body size, reproduction, behavior, DNA methylation, coloration, and bone production have occurred, among others (Price 1984; Jensen and Wright 2014; Johnsson *et al.* 2015a, 2015b, 2016; Johnsson, Henriksen, Fogelholm, *et al.* 2018; Johnsson, Henriksen, Höglund, *et al.* 2018; Fogelholm *et al.* 2019, 2020; Höglund *et al*. 2020). In addition to these, the comb of the chicken has been under strong selection, both for size and a variety of different large effect morphological changes derived from single-gene mutations. These mutations include such phenotypes as the pea comb (Wright *et al.* 2009), rose comb (Imsland *et al.* 2012), and duplex comb (Dorshorst *et al.* 2015) and were used by Bateson as the first examples of Mendelian inheritance in animals and of 2-gene epistatic interactions (Bateson and Mendel 1913). Despite the interest in the genetics of the comb, more complex morphological evaluations of the chicken comb and the genes underpinning such morphology have yet to be performed. The classic single comb consists of several different morphological features including number of finger-like protrusions (comb fingers) and posterior paddle-like structures, which together form the general comb shape.

In chickens, the comb serves as a sexual ornament, which plays a role not only in males for social rank, mate choice, and heat regulation but is also linked to female egg production, fecundity, bone mass, and tarsus length (Cornwallis and Birkhead 2007a, 2007b; Wright *et al.* 2008; Johnsson *et al.* 2014). Therefore, larger combs result in higher egg production and are the target of anthropogenic selection in captivity as well as sexual selection in nature. In this regard, it has been shown that females prefer males with larger combs that will increase viability (Parker 2003) and assure males with increased bone reserves (Wright *et al.* 2008; Johnsson *et al.* 2014), whereas males prefer females with larger combs, as this signals superior maternal investment (Pizzari *et al.* 2003; Cornwallis and Birkhead 2007a, 2007b) and increased reproductive output (Wright *et al.* 2008). The high heritability rates of comb variation in chickens highlight a strong genetic component for this trait (Johnson *et al.* 1993). Indeed, several studies have reported that quantitative trait loci (QTL) found to be associated to different aspects of comb morphology, size, and mass (Wright *et al.* 2009; Johnsson *et al.* 2012, 2014; Sun *et al.* 2015; Shen *et al.* 2016). Furthermore, gene expression analysis from bone marrow tissue provided a few examples of bone allocation genes that colocalize in these comb-related regions, supporting a pleiotropic effect of higher egg production associated to larger combs (Johnsson *et al.* 2012, 2014). With regard to the phenotype, most of these studies concentrated on comb length, comb height, and comb weight with the exception of Moro *et al.* (2015), who analyzed the impact of DNA copy number variants on geometric morphometric land-mark data.

The use of classic morphological measurements in QTL analyses has proven to be fruitful thus far, however, different components of shape, captured using geometric morphometric approaches, are less utilized and often reveal novel genomic regions being associated to the studied trait (e.g. Takahara and Takahashi 2015). In the present study, we add chicken comb shape outline and novel gene expression data to previously published DNA sequence and gene expression datasets (Johnsson *et al.* 2012, 2014), to find genomic and transcriptomic associations to various aspects of comb morphology and size. In doing so, we confirmed the presence of a large effect QTL associated to a size-related comb trait (surface area) but also identified novel QTL associated to comb shape (outline) and comb finger number. We show that digitized photographs of highly variable chicken combs can be used to extract estimate trait measures for subsequent QTL analyses. These results add on to an ever-growing understanding of the comb serving as a sexual ornament in the chicken system.

## Materials and methods

### Advanced intercross lines and genetic markers

The chicken combs used in this study were obtained from an F8 population generated by crossing a line of selected White Leghorn maintained from the 1960s and a population of Red Junglefowl obtained from Thailand. A genetic map, consisting of 651 informative SNP markers, was used in conjunction with 326 F8 individuals (201 males and 125 females) for the interval mapping. These markers resulted in a map of length ∼9,200 cM, with an average marker spacing of ∼16 cM. Of these markers, 551 were fully informative (differentially fixed between the parental populations), with the markers selected so as to be evenly spread over the genome (as reflected in the average marker spacing) (Wright *et al.* 2010, 2012). This marker density is greater than the recommended 20–30 cM marker intensity for QTL analyses (Darvasi and Soller 1994). A more detailed description of the intercross can be found in Johnsson *et al.* (2012).

### Gene expression

RNA was isolated from tissue at the base of the comb for 39 male individuals. Adult comb base tissue was used, as previously when assessing quantitative differences in comb size adult comb base tissue showed strong correlations with comb size (Johnsson *et al.* 2012). Double-stranded cDNA was labeled and hybridized to NimbleGen 12 × 135k custom gene expression arrays (Roche NimbleGen). Gene expression data used for the interval mapping can be found in Supplementary Table 1. A detailed description of how the gene expression data was generated and processed can be found in Johnsson *et al.* (2014), who investigated a subset of probe sets associated with the studied comb mass QTL.

### Comb phenotypes

**Fig. 1.**
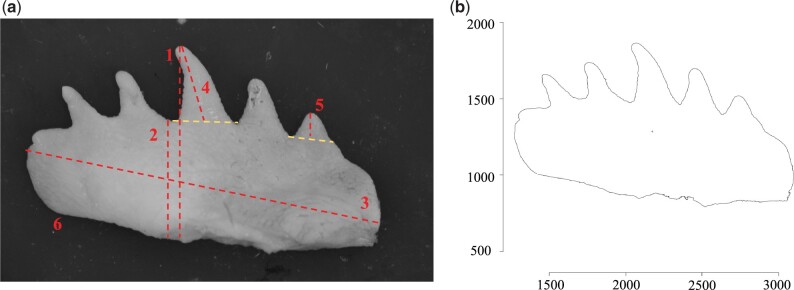
a) Example of comb with 5 fingers and phenotypic measures used in the QTL interval mapping analysis (1, comb height max; 2, comb height min; 3, comb length; 4, max finger length; 5, min finger length; 6, comb area). b) Extracted outline of this same comb presented in coordinate space.

Chicken combs were dissected and stored under −20°C prior to analysis. To extract comb phenotype data, a Nikon digital camera (D3100) with an 85-mm lens was used to generate digital images. Combs were laid down flat and pictures were taken under the same conditions for each sample within a white photo-tent and along-side a white color checker that was used as a length scale. Comb length, height, area, and the number and size of fingers were measured using the *measure and selection* tools in IMAGEJ v1.51 (Schneider *et al.* 2012) (Fig. 1). To extract comb outline measures, images were first converted to black and white and then inverted so as to have the combs as black objects on a white background prior to being imported into the *R* package MOMOCS, which was then used to extract comb outlines as *x* and *y* coordinates (Bonhomme *et al.* 2014). Phenotype measurements and genotypes used in this study can be found in the QTL cross file used for the QTL interval mapping (Supplementary Table 2).

Comb outlines were centered, scaled, and aligned before performing a final generalized Procrustes alignment. Procrustes alignment and Fourier analysis require equal numbers of coordinates for each outline; hence, all outlines were interpolated to select 16,570 coordinates in each (derived from the comb with the highest number of coordinates). Fourier coefficients were derived by running an Elliptical Fourier Analysis (EFA) on aligned outline data in MOMOCS, which did not require further normalization. In EFA, shape is defined with Elliptic Fourier Descriptors, i.e. number of cosine waves (harmonics) required to describe variation of the comb outline (Campana and Casselman 1993; Crampton 1995). In this case, 26 harmonics were sufficient to accurately describe comb outlines (>99% harmonic power as estimated using MOMOCS) and produced 200 coefficients, which were further analyzed by principal component analysis (PCA). The first principal component (PC1) explained ∼35%, while the second principal component (PC2) explained ∼14% of the variation. The highly variable comb shapes resulted in PC axis-specific descriptions of different aspects to comb shape but to utilize axes with the highest proportion of variation explained, we used only PC1 and PC2 (Supplementary Fig. 1).

### QTL and eQTL analyses

The R package, R/qtl, was used to estimate LOD scores of individual markers to phenotypic measurements based on additive and dominance models with sex, batch, population structure, and weight as fixed differences (Broman *et al.* 2003; R Development Core Team 2013). These were run additionally using sex as an interacting covariate to account for QTL varying between the sexes. LOD score significance thresholds were estimated via permutation tests for each phenotype, by shuffling individual labels and calculating LOD scores 1,000 times to produce null distributions. Scores were considered significant if they lay above the 0.95th quantile of the randomized distributions and suggestive if they laid above the 0.80th quantile (corresponding *P* values of 0.05 and 0.2, respectively). A suggestive threshold was used as per (Lander and Kruglyak 1995), though we used a 20% genome-wide threshold, which is slightly more stringent than the 1 false positive per scan method utilized by Lander and Kruglyak.

Gene expression data for over 35 thousand probe sets were obtained using a 135K-Affimetrix micro array and were used as phenotypes in an interval mapping analysis. Here, batch was used as a fixed factor in the model. LOD thresholds were estimated as above, using permutations. As the genomic positions of the probe sets are known, *cis* (or local) eQTL were assigned if the probe set lay within 50 cM of the marker exhibiting the highest LOD score. All others were assigned as trans eQTL. Confidence intervals for QTL and eQTL were estimated using a 1.8 LOD drop method (i.e. where LOD scores decrease by 1.8 in both directions of the LOD peak) and is comparable to a 95% confidence interval for intercross populations (Manichaikul *et al.* 2006).

### Overlapping confidence intervals between QTL and eQTL

To establish which QTL and eQTL contained overlapping confidence intervals, we used the GENOMICS RANGES package in R (Lawrence *et al.* 2013). Overlaps were conducted using the genomic positions on the *galgal6* version of the chicken genome. Genomic positions were extracted by using the closest marker to the start and end of each QTL and eQTL confidence interval. Furthermore, gene expression of identified eQTL was correlated with overlapping QTL phenotype measurements using to *cor.test* function with Pearson’s method in R. To test if overlaps between QTL and eQTL were enriched (i.e. significantly higher than expected by chance), we conducted a permutation test by selecting 10 random regions (representing QTLs with average QTL CI size) and 1,184 random regions (representing eQTLs with average eQTL CI size) from a range the size of the chicken genome and counting the number of overlapping regions between these 2 sets. This was repeated 10,000 times to produce a random distribution against which the observed value was compared.

### Overlapping QTL confidence intervals with selective sweeps

To additionally validate the biological significance of our QTL, we tested whether any of our findings overlapped with previous detections of selective sweeps (Qanbari *et al.* 2019), which were identified by comparing Red Junglefowl genomes to those of several domestic modern chicken breeds. As in the original study an older version of the chicken genome assembly was used (*Galgal4*), we converted this data to match that of the newest genome assembly (*Galgal6*) with the NCBI REMAP tool and used the GENOMIC RANGES R package to identify overlapping regions between detected QTL confidence intervals and selective sweep regions (Supplementary Table 9 from Qanbari *et al.* 2019). A permutation test as detailed above was conducted to test for enrichment in overlaps between selective sweeps and QTL. Here, 645 random regions, representing selective sweeps with average selective sweep length, were selected from a hypothetical genome the size of the chicken genome, and were overlapped with random QTL regions (see above) 10,000 times.

## Results

### Phenotypic measurements of the chicken comb

**Fig. 2.**
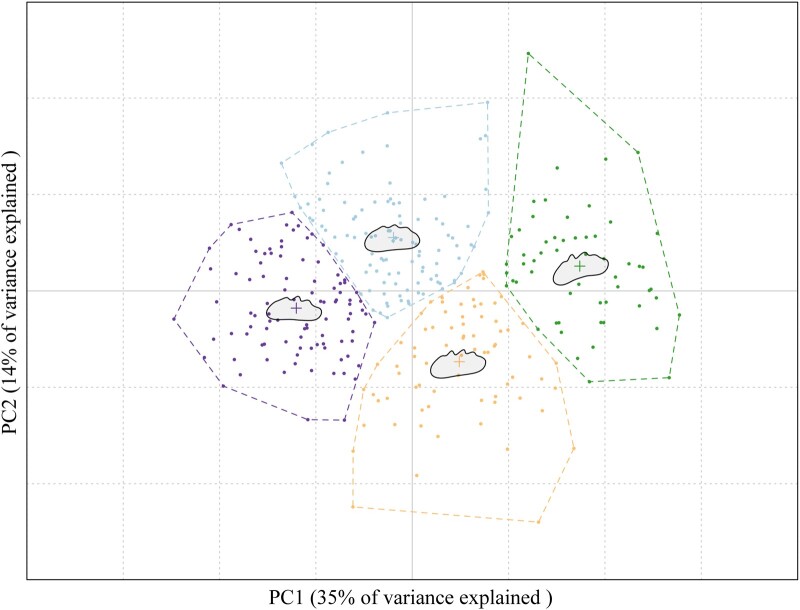
PCA plot showing PC1 vs. PC2 scores of each sample based on its comb outline coordinate data. PC1 is conspicuous with regards to anterior comb shape, and PC2 distinguishes between a comb having more and bigger fingers (bottom) vs. less and smaller fingers (top). Groupings are arbitrary and based on PC axes (representing high and low PC1 and PC2 scores) as implemented in the MOMOCS package.

Phenotypic measurements of finger number, finger size, surface area, height, width, and PCA of outline coordinate data of all 326 F8 individuals can be found within the cross file used in R/qtl for interval mapping (Supplementary Table 2). PCA of comb outline data revealed the presence of 2 main shape components along PC axes 1 and 2, with PC1 targeting the anterior comb shape and PC2 targeting the dorsal “roughness” caused by comb fingers (Fig. 2). For a representation of explained variation by PC axes 1–10, see Supplementary Figs. 1 and 2, where mean shapes with standard deviations for each PC axis, as well as variation explained by each are presented.

### Comb QTL

QTL interval mapping results are presented in Table 1. In total, we identified 10 significant and/or suggestive QTL associated to the various trait measurements (3 to comb area, 1 to comb outline, 1 to finger number, 1 to finger length, 2 to comb length and 2 to comb height max). The QTL with largest effect identified in this study is located on chromosome 3 and is associated to comb area and height (i.e. size-related traits; Table 1). Additional information on QTL results, including genomic positions and genes present within confidence intervals, can be found in Supplementary Table 3.

**Table 1.** QTL interval mapping results showing 10 significant and suggestive QTL found associated to various chicken comb phenotypes measured in this study.

Phenotype	Marker	Chr	Pos	LOD	*P*-value	CI S	CI E	Add. effect	Add. SE	Dom. effect	Dom. SE	%Var
Area	chr3_16000000	3	148	9.70	0.006	136	155	887.5	149.64	−112.79	203.47	1.68
Area	Gg_rs15491847	1	1,965	4.40	0.11	1947	1983	225.7	55.75	268.58	79.78	0.79
Area	GG_rs14304199	27	96	7.02	0.17	83	117	1414.1	260.34	766.73	505	1.24
PC1 outline	Gg_rs15752067	6	1	5.29	0.07	0	20	1.9e−04	3.4e−04	−1.9e−03	4.6e−04	5.78
finger no.	2_26017380	2	285	3.05	0.16	237	348	0.2597	0.07	−0.067	0.095	3.07
Max finger len.	Gg_rs14133982	2	67	7.34	0.046	53	81	−4.22	1.41	−12.99	2.63	1.87
Length	Gg_rs13965220	1	1,955	4.19	0.09	1936	1982	5.32	1.32	6.34	1.92	0.76
Length	Gg_rs16155378	2	1,162	3.10	0.11	1140	1175	−2.99	0.89	2.19	1.21	0.57
Height max	chr3_16000000	3	148	10.57	0.003	144	156	8.19	1.57	1.78	2.13	2.11
Height max	Gg_rs14026230	11	111	5.79	0.14	99	121	−2.48	1.69	9.44	2.61	1.19

CI S, CI start; CI E, CI end; Add. and Dom. effect, additive and dominance effects along with standard errors; % Var, percent variation explained.

### Gene expression eQTL

We identified 1,184 gene expression eQTL in comb base tissue (660 *trans* and 524 *cis*). All significant and suggestive cis and trans eQTL can be found in Supplementary Table 4.

### Overlaps between QTL and eQTL

In total, confidence intervals of 98 (out of 1,184) eQTL were found overlapping those of the 10 QTL. Of these, 47 (19 *cis*; 28 *trans*) overlapped with comb area (60% *trans*), 6 (5 *cis*; 1 *trans*) with comb height max (17% *trans*), 23 (11 *cis*; 12 *trans*) with comb length (52% *trans*), 15 (6 *cis*; 9 *trans*) with finger number (60% *trans*), 2 (2 *cis*) with max finger length (0% *trans*), and 5 (5 *trans*) with PC1 scores of our outline analysis (100% trans) (Supplementary Table 5). Here, a QTL associated to finger number overlapped with *CP450CC24*, a gene tied to bone regeneration. Regions where confidence intervals of our detected QTL and eQTL overlap can be found in Supplementary Table 5. To prove significance, a higher number of overlapping regions were found than by chance alone, showing overlap enrichment between eQTL and QTL identified in this study (permutation test; 10,000 iterations; 1-tailed *P*-value <0.007). Only significant correlations between gene expression and QTL phenotype are presented in Table 2 and only include correlations with comb surface area. All correlations between gene expression and QTL phenotypes can be found in Supplementary Table 5.

**Table 2.** Overlaps between eQTL and QTL confidence intervals.

eQTL phenotype	eQTL CI	QTL phenotype	QTL CI	QTL/eQTL correlation	Correlation *P*-value
ENSGALT00000005938_CPAMD8	chr3:140–160	Comb area	chr3:136–155	0.605	0.00175
ENSGALG00000009078_GALNT14	chr3:137–177	Comb area	chr3:136–155	0.595	0.00214
ENSGALT00000014770_GALNT14	chr3:140–172	Comb area	chr3:136–155	0.595	0.00214
603866551F1	chr3:116–159	Comb area	chr3:136–155	0.576	0.00322
ENSGALT00000041038_Q6RUW0_CHICK	chr3:137–165	Comb area	chr3:136–155	0.572	0.00346
ENSGALT00000011900_CILP	chr3:138–166	Comb area	chr3:136–155	0.568	0.00378
603867953F1	chr3:143–157	Comb area	chr3:136–155	0.566	0.00396
603597474F1	chr3:147–160	Comb area	chr3:136–155	0.552	0.00514
ENSGALT00000018417_C0L7M6_CHICK	chr3:141–165	Comb area	chr3:136–155	0.535	0.00704
ENSGALT00000039270_LOC768788	chr3:101–142	Comb area	chr3:136–155	0.525	0.00837
ENSGALT00000025114_HTRA3	chr3:138–163	Comb area	chr3:136–155	0.521	0.00908
ENSGALT00000013983_GPR1	chr3:137–167	Comb area	chr3:136–155	0.517	0.00975
ENSGALT00000014374_HAO1	chr3:105–176	Comb area	chr3:136–155	0.503	0.01226
ENSGALT00000007955_P87363_CHICK	chr3:136–162	Comb area	chr3:136–155	0.488	0.01566
ENSGALG00000015624_VCAN	chr3:137–163	Comb area	chr3:136–155	0.472	0.0199
NM_001030649_EIF4A3	chr3:122–161	Comb area	chr3:136–155	−0.438	0.03242
ENSGALG00000016954_RGCC	chr3:140–173	Comb area	chr3:136–155	0.427	0.03756

Only significant correlations between eQTL gene expression and QTL phenotype are included. Note, only correlations with comb area were significant.

### Overlaps between QTL and selective sweeps

In total, 99 [out of 645 selective sweep regions identified in Qanbari *et al.* (2019)] were found within confidence intervals of detected QTLs from this study. These included 23 within comb length and 78 within finger number QTL (Supplementary Table 6). A comparison of the observed number of overlaps between sweep regions and QTL with the number of overlaps between random regions showed a high degree of overlap enrichment (permutation test; 10,000 iterations; 1-tailed *P*-value <0.0001).

## Discussion

We identified several QTL associated with comb size, comb finger number, and comb outline coordinate data. The confidence intervals of these QTL overlapped with those of several eQTL, among which a gene related to bone regeneration was identified. However, significant correlations between gene expression and trait measurements were only found in the case of comb area. These confirm and are a continuation to previous associations found between comb, reproduction, and several bone allocation related genes (Johnsson *et al.* 2014), further highlighting how this trait may function as a sexual ornament in the chicken system. These bone-related candidate genes are of particular relevance, given that cartilage is a precursor to bone formation (Johnsson *et al.* 2012, 2014). Similarly, strong phenotypic and genetic correlations have been found in this intercross between comb mass, bone allocation, and fecundity.

A novel QTL on chromosome 6 was found associated to PC1 of our comb outline coordinate data and 2 novel QTLs on chromosome 2 were found associated to finger number and finger size. To the best of our knowledge, no genomic regions were found associated to these comb traits before. The comb shape outline QTL overlapped with 3 annotated eQTL (*DCDC2*, *TNFRSF1*, and *RXFP1*). The encoded protein of *RXFP1*, relaxin receptor 1, has strong effects on sperm motility in males, and parturition in females (Park *et al.* 2005; Ferlin *et al.* 2012). Some biological processes associated to *DCDC2* are cilium assembly, neuron morphogenesis, and sensory perception of sound (Grati *et al.* 2015; Girard *et al.* 2016). Mutations in *TNFRSF1* underlie tumor necrosis factor receptor-associated periodic syndrome (*TRAPS*) and with multiple sclerosis in human patients (Ottoboni *et al.* 2013). eQTL-QTL overlaps were largely not biased between cis and trans eQTL, except in the case of comb shape where only trans eQTL were present. This adds on to the validity of this finding as they were found associated to gene expression patterns within the same genomic region. Further, several eQTL had overlapping confidence intervals with finger number and size QTL on chromosome 2. Of notable interest, *WIPF3* codes for the WAS/WASL protein family, which are corticosteroids and may potentially be involved in spermatogenesis [estimated by gene similarity on Uniprot.org and indicated in Roy *et al.* (2017)]. Interestingly, the gene *CP450CC24*, within the overlap region with finger number, has been associated to bone regeneration specifically in studies conducted on White Leghorn chickens (Seo and Norman 1997). In addition, the CI of finger number and comb length QTL overlapped with several selective sweep regions identified in Qanbari *et al.* (2019), indicating that comb, bone, and reproductive investment traits along with these genomic regions are likely experiencing enhanced selection in domesticated chicken breeds. One caveat with all the above gene candidates is that these are found by correlational analyses, and full functional assays are required before one can be certain that these do indeed modify comb morphology.

In a previous study, a large effect QTL was found associated to comb weight (Johnsson *et al.* 2012). Moreover, this QTL was found in close vicinity to 2 genes, *HAO1* and *BMP2*, which affect bone strength and egg laying capacities. Here, we find this same region associated to comb surface area with the expression of *HAO1* being significantly correlated to comb surface area measurements. This result is not surprising as comb weight was positively associated to *HAO1* expression in a previous study (Johnsson *et al.* 2014) and comb surface area is expected to be correlated with comb weight.

To summarize, we present novel putative QTL associated to different aspects of comb shape, namely, finger number and size and shape outline coordinate data. We demonstrate a significant enrichment in the overlap between gene expression probe sets and QTL with the implication of several genes being associated to QTL phenotypes, including *RXFP1* and *CP450CC24*. Furthermore, overlapping regions between selective sweeps tied to domestication and comb length and finger number QTL would suggest that the entailed genes are putatively under selection. This would imply that these phenotypes and associated genomic regions have undergone or are currently under anthropogenic selection. Whether these same genomic regions are under selection in wild populations warrants further investigation by conducting genome-wide association studies and selective sweep scans in feral chicken populations.

## Data availability

Genotype and gene expression data are available at Dryad: https://doi.org/10.5061/dryad.bs275. Chicken comb phenotype data generated in this study are available in supplementary materials at figshare: https://doi.org/10.25387/g3.20103158.
